# Measuring Sperm DNA Fragmentation and Clinical Outcomes of Medically Assisted Reproduction: A Systematic Review and Meta-Analysis

**DOI:** 10.1371/journal.pone.0165125

**Published:** 2016-11-10

**Authors:** Maartje Cissen, Madelon van Wely, Irma Scholten, Steven Mansell, Jan Peter de Bruin, Ben Willem Mol, Didi Braat, Sjoerd Repping, Geert Hamer

**Affiliations:** 1 Department of Obstetrics & Gynaecology, Jeroen Bosch Hospital, ‘s-Hertogenbosch, The Netherlands; 2 Center for Reproductive Medicine, Academic Medical Center, University of Amsterdam, Amsterdam, The Netherlands; 3 Department of Molecular and Cell Biology, University of California, Berkeley, California, United States of America; 4 The Robinson Institute/School of Paediatrics and Reproductive Health, University of Adelaide, Adelaide, Australia; 5 Department of Obstetrics and Gynaecology, Radboud University Medical Center, Nijmegen, The Netherlands; Universite Blaise Pascal, FRANCE

## Abstract

Sperm DNA fragmentation has been associated with reduced fertilization rates, embryo quality, pregnancy rates and increased miscarriage rates. Various methods exist to test sperm DNA fragmentation such as the sperm chromatin structure assay (SCSA), the sperm chromatin dispersion (SCD) test, the terminal deoxynucleotidyl transferase mediated deoxyuridine triphosphate nick end labelling (TUNEL) assay and the single cell gel electrophoresis (Comet) assay. We performed a systematic review and meta-analysis to assess the value of measuring sperm DNA fragmentation in predicting chance of ongoing pregnancy with IVF or ICSI. Out of 658 unique studies, 30 had extractable data and were thus included in the meta-analysis. Overall, the sperm DNA fragmentation tests had a reasonable to good sensitivity. A wide variety of other factors may also affect the IVF/ICSI outcome, reflected by limited to very low specificity. The constructed hierarchical summary receiver operating characteristic (HSROC) curve indicated a fair discriminatory capacity of the TUNEL assay (area under the curve (AUC) of 0.71; 95% CI 0.66 to 0.74) and Comet assay (AUC of 0.73; 95% CI 0.19 to 0.97). The SCSA and the SCD test had poor predictive capacity. Importantly, for the TUNEL assay, SCD test and Comet assay, meta-regression showed no differences in predictive value between IVF and ICSI. For the SCSA meta-regression indicated the predictive values for IVF and ICSI were different. The present review suggests that current sperm DNA fragmentation tests have limited capacity to predict the chance of pregnancy in the context of MAR. Furthermore, sperm DNA fragmentation tests have little or no difference in predictive value between IVF and ICSI. At this moment, there is insufficient evidence to recommend the routine use of sperm DNA fragmentation tests in couples undergoing MAR both for the prediction of pregnancy and for the choice of treatment. Given the significant limitations of the evidence and the methodological weakness and design of the included studies, we do urge for further research on the predictive value of sperm DNA fragmentation for the chance of pregnancy after MAR, also in comparison with other predictors of pregnancy after MAR.

## Introduction

Traditionally, the diagnosis of male subfertility is based upon the analysis of semen volume and sperm concentration, motility and morphology. Although there is a direct relationship between semen quality and pregnancy rates both in natural conception and after medically assisted reproduction (MAR), there is no definite predictive threshold for success for conventional semen parameters [1–4]. Conventional semen analysis does not assess all aspects of the function of testis and sperm quality. New tests for predicting the chance of pregnancy would be clinically useful. There have been attempts to propose sperm DNA fragmentation as such a new test for male reproductive capability [5].

The integrity of our genome is continuously challenged by endogenous metabolic by-products and exogenous factors. Depending on variables like cell type, cell cycle stage and the type of DNA damage, a cell has several ways to repair damaged DNA and inaccurate repair can have different consequences [6,7]. While our somatic bodies inevitably die of old age or disease, the germ line has to maintain sufficient DNA integrity to pass on our genome to forthcoming generations. DNA double-strand breaks (DSBs) are endogenously induced during spermatogenesis; first during meiosis, to facilitate the formation of meiotic crossovers, and second during spermiogenesis, when the chromatin of the haploid round spermatids is compacted by the replacement of histones by protamines [8,9]. Furthermore, the sperm may accumulate DNA damage and fragmentation during maturation and storage in the epididymis [10–12]. Other causes of sperm DNA fragmentation can be defective apoptosis, excessive reactive oxygen species (ROS) production and decreased seminal antioxidants [13–23]. Also toxic effects of drugs, cigarette smoking, pollution, and factors as xenobiotics, high testicular temperature (fever, varicocele) and advanced age have been associated with increased sperm DNA damage [24–28].

Recent studies have highlighted the significance of sperm DNA integrity as an important factor that affects functional competence of the sperm. Therefore the detection of sperm DNA fragmentation could be clinically useful as part of fertility workup [29]. For this purpose, several techniques that measure DNA fragmentation are available and have been evaluated in separate studies. In this systematic review we will assess the sperm chromatin structure assay (SCSA), the sperm chromatin dispersion (SCD) test, the terminal deoxynucleotidyl transferase mediated deoxyuridine triphosphate nick end labelling (TUNEL) and the single cell gel electrophoresis (Comet) assay.

The SCSA bases its results on (1) the DNA fragmentation index (DFI), which is the percentage in the sample that have measurable increased red fluorescence due to acridine orange attaching to a single strand portion of DNA at sites of DNA strand breaks and then collapsing into a crystal that produces a metachromatic shift to red fluorescence under exposure to blue light and (2) the percentage of high DNA stainability (HDS), which is due to excess histones and proteins other than protamines that prevent full condensation of the sperm chromatin [30–32].

The SCD test, also known as Halo Sperm assay, estimates the level of DNA fragmentation indirectly by quantification of the amount of nuclear dispersion/halo after sperm lysis and acid denaturation to remove excess nuclear proteins [33,34].

The principle of TUNEL involves labelling of the 3′-ends of single- and double-strand breaks with biotinylated dUTPs. The incorporated labelled nucleotides can be quantified by flow cytometry or (fluorescence) microscopy to determine the number of (apoptotic) sperm cells containing fragmented DNA [31]. However, double strand DNA can have breaks with no exposed 3’OH end and thus not being labelled by the TUNEL assay.

The Comet assay quantifies the shape of the single cell nuclei after gel electrophoresis. Small fragmented DNA has a faster rate of migration towards the anode in an electrophoretic field (tail region) as compared to larger non-fragmented DNA (head region), leading to a typical comet shape [30,31].

Using these tests, the percentage of sperm with fragmented DNA was shown to be comparable in idiopathic subfertile men with normal sperm parameters and in subfertile men with abnormal sperm parameters, and significantly higher in both these groups in comparison to fertile controls [35–37]. However, we do not know whether MAR helps to overcome the negative effects of DNA damage on the chance of pregnancy. We therefore performed a systematic review and meta-analysis to assess the value of measuring sperm DNA fragmentation in predicting the ongoing pregnancy chance after in vitro fertilization (IVF) or intracytoplasmic sperm injection (ICSI). 

## Materials and Methods

### Search and selection strategy

The electronic databases Pubmed, Embase, Cochrane and CINAHL were searched from inception (September 1967) to January 2016 for articles which described sperm DNA fragmentation tests and outcome after MAR. The Medical Subject Headings terms and/or text words that were used in our search can be found in the appendix. We also manually reviewed the bibliographies of retrieved original papers and review articles. We used the preferred reporting items for systematic review and meta-analysis checklist (PRISMA) while conducting this study (S1 Fig).

Titles and abstracts of all identified studies were screened and the full paper of the preselected articles was read by two researchers (S.M. and M.C.). Both researchers extracted the data from the article independently by using standardized data extraction forms. If 2x2 tables could be constructed the study was selected for final inclusion. In the 2x2 tables, the numbers of pregnant and non-pregnant women for different sperm DNA fragmentation cut-off values were recorded. Any disagreement between the two researchers was resolved through discussion or by consultation with a third researcher (I.S.).

### Eligibility criteria

All studies investigating the effect of sperm DNA fragmentation detected by the SCSA, the SCD test, the TUNEL assay or the Comet assay on the outcome of IVF and/or ICSI were considered eligible for inclusion. The search was restricted to studies in humans. Studies were excluded if they had no original data available for retrieval and duplicate publications were also excluded. Studies that included cycles with donor oocytes and experiments that asses sperm DNA fragmentation in specific male factor pathologies e.g. azoospermia and co-intervention experiments e.g. antioxidant treatment were excluded from analysis.

The primary study outcome was ongoing pregnancy (defined as the presence of a living intrauterine fetus on transvaginal ultrasonography (TVU) at the 12th week of gestation). Other study outcomes were clinical pregnancy (defined as the presence of a gestational sac on TVU or other definitive clinical signs) and live birth (defined as a live-born baby ≥ 24 weeks of gestation). All outcomes were reported per cycle.

### Quality assessment

Each selected study was scored for their relevance and methodological quality by using the QUADAS 2 (Quality Assessment of Diagnostic Accuracy Studies) checklist [38]. Furthermore the following characteristics of the studies were taken into consideration: data collection method (prospective of retrospective), study design (cohort or randomized controlled trial (RCT)) and study population.

### Statistical analysis

#### Hierarchical summary receiver operating characteristic

In order to evaluate the overall accuracy, including the whole range of possible thresholds, we used hierarchical summary receiver operating characteristic (HSROC) plots to display the results of individual studies in a ROC space, each study being plotted as a single sensitivity-specificity point. Reported estimates for sensitivity and specificity from different studies may be based on different positivity thresholds (explicitly due to cut-off values used, or implicitly related to the assessment method or device used). If there are multiple thresholds reported in one study, we chose the threshold that was most comparable to the others. Based on the binomial distributions of the true positives and true negatives we calculated a summary point, with a 95% confidence interval (CI) and predictive interval by using STATA version 14 (Stata-Corp, College Station, Texas, USA). As recommended for meta-analysis of diagnostic accuracy studies [39], we used hierarchical models to obtain summary estimates of sperm DNA fragmentation test in terms of ability to discriminate between men with lower and higher probabilities of pregnancy. Separate HSROC curves for IVF and ICSI were performed when sufficient studies were available.

An area under the curve (AUC) of 1 implies perfect discrimination, whereas an AUC of 0.5 means that the test does not discriminate at all [40]. For this review, a test is considered to have a poor predictive accuracy if the AUC lies between 0.50 and 0.70. An AUC between 0.70 and 0.80 represents a fair predictive accuracy, and an AUC above 0.80 represents a good predictive accuracy.

In cases where insufficient data was available to perform HSROC analyses for clinical pregnancy, (ongoing) pregnancy or live birth independently, different pregnancy outcomes were combined when minimal differences in sensitivity and specificity were found. When minimal differences in sensitivity and specificity were found, studies with different timing of the sperm DNA fragmentation test (pre- and post-wash) were combined.

#### Pooled sensitivity and specificity

According to the bivariate method [41], sensitivity and specificity with 95% CI were calculated and displayed in a forest plot. Sensitivity and specificity of original studies were pooled with STATA, using a random effect model. This model was chosen for explicating the heterogeneity between the included studies and to estimate the between-study variance. The amount of heterogeneity was quantified by using the I^2^ statistic, which represents the percentage of total variability across the studies that is due to heterogeneity instead of chance. Moderate heterogeneity is defined as a value < 50% [42].

#### Meta-regression

Meta-regression analysis was performed with type of fertility treatment as covariable to determine if differences in fertility treatment affected the estimated effect of sperm DNA fragmentation. The meta-regression analyses were performed using STATA subroutine MIDAS. If the p-value was < 0.05, results were considered to indicate statistical significance.

## Results

### Systematic search, selection and data extraction

**Fig 1:**
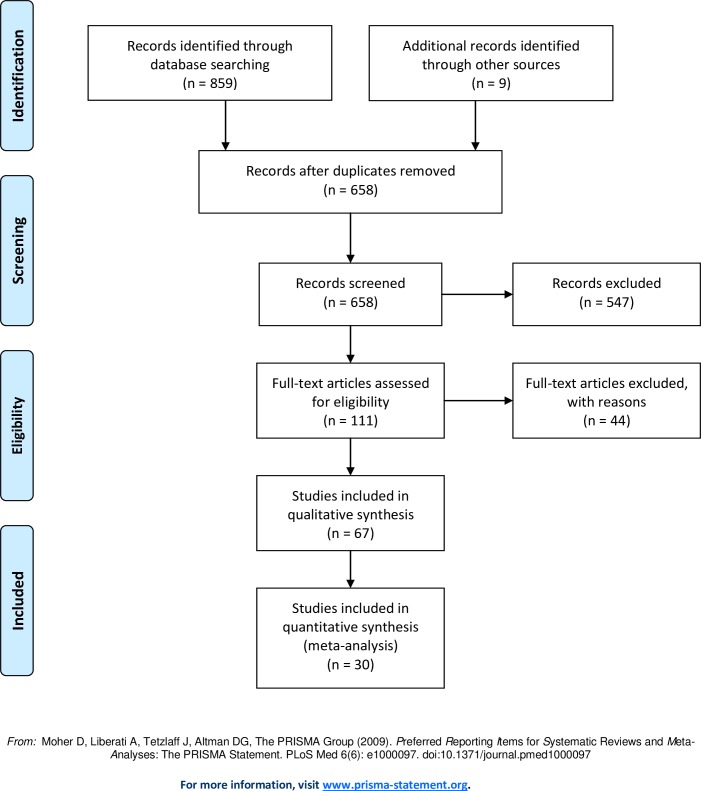
Flowdiagram of search and selection strategy in a systematic review and meta-analysis of sperm DNA fragmentation tests and pregnancy rates after MAR. Legend: not applicable.

The electronic search resulted in 859 hits. Following the removal of duplicates and the addition of studies by hand-search and screening of abstracts, 111 studies were identified to be potentially eligible for inclusion (Fig 1).

After reading the manuscripts and assessing the inclusion criteria and methodological quality, 67 studies were found that evaluated the association between sperm DNA fragmentation and pregnancy after IVF or ICSI and 30 studies were eligible for final inclusion into the diagnostic meta-analyses. We excluded 81 studies for the reasons shown in Table 1.

**Table 1:** List of studies excluded from the meta-analysis.

Treatment with IUI method	Alkhayal et al., 2013; Duran et al., 2002; Muriel et al., 2006; Yang et al., 2011 [43–46]
Inappropriate inclusion criteria	Dar et al., 2013; Gosalvez et al., 2013; Greco et al., 2005; Morris et al., 2002; Nunez Calonge et al., 2012; Wang et al., 2012 [47–52]
Overlapping data	Bungum et al., 2004; Bungum et al., 2008; Henkel et al., 2003; Jiang et al., 2011; Larson et al., 2000; Simon et al., 2013 [53–58]
Outcome fertilization rate or biochemical pregnancy	Cebesoy et al., 2006; Claassens et al., 1992; Daris et al., 2010; Host et al., 2000; Lopes et al., 1998; Marchetti et al., 2002; Pregl Breznik et al., 2013; Sadeghi et al., 2009; Sun et al., 1997 [36,59–66]
Use of assays not included in the systematic review	Abu-Hassan et al., 2006; Angelopoulos et al, 1998; Chi et al., 2011; Duran et al., 1998; Edwards et al., 2015; Filatov et al., 1999; Hammadeh et al., 1996; 1998; 2001; 2001; Hoshi et al., 1996; Jiang et al., 2011; Karydis et al., 2005; Katayose et al., 2003; Larazos et al., 2011; Sakkas et al., 1996; Tavares et al., 2013; Tomlinson et al., 2001; Virant-Klun et al., 2002; Zhang et al., 2008; Zini et al., 2005 [67–87]
Data not extractable because of language	Bufang et al., 2011; Fang et al., 2011; Xi et al., 2016; Yang et al., 2013 [88–91]
Insufficient data to construct 2x2 table	Avendano et al., 2010; Bakos et al., 2008; Benchaib et al., 2003; Caglar et al., 2007; Garolla et al., 2015; Gu et al., 2009; 2011; Hammadeh et al., 2006; 2008; Irez et al., 2014; Jin et al., 2015; Kennedy et al., 2011; Khalili et al., 2014; Lewis et al., 2004; Li & Jiang, 2011; Lopez et al., 2013; Meseguer et al., 2011; Nasr Esfahani et al., 2008; Nicopoullos et al., 2008; Nijs et al., 2009; 2011; Rama Raju et al., 2012; Saleh et al., 2003; Sanchez-Martin et al., 2013; Sharbatoghli et al., 2012; Smit et al., 2010; 2010; Tarozzi et al., 2009; Tavalaee et al., 2009; Tomsu et al., 2002; Velez de la calle et al., 2008 [92–122]

### Descriptive review

We found 21 studies reported on the SCSA [103,110–112,114,117,118,123–136], 18 studies on the SCD test [97,98,101,102,106–109,113,115,116,120,122,137–141], 18 studies reported on the TUNEL assay [92–95,96,99,100,104,119,134,142–149] and seven studies reported on the alkaline Comet assay [95,105,121,134,150–152].

### Studies selected for diagnostic meta-analysis

**Fig 2:**
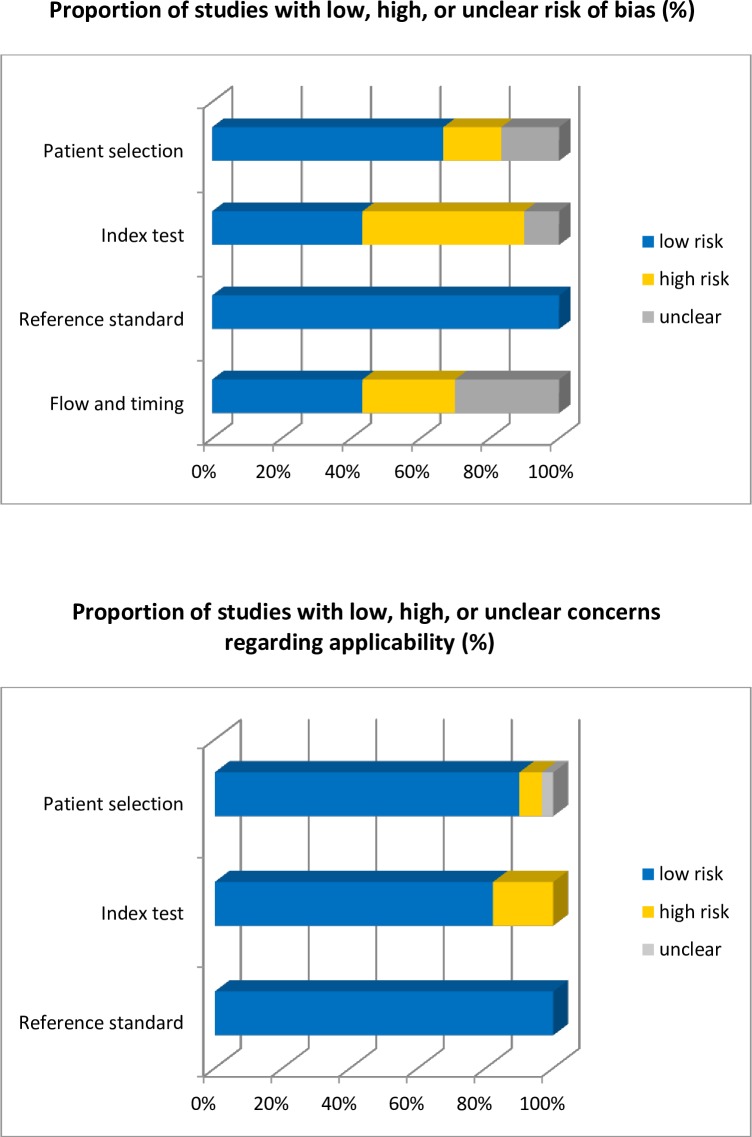
Overall risk of bias in meta-analysis. This figure illustrates the overall risk of bias in the meta-analysis. The horizontal axis represents the number of studies included. The color of the bars represent the risk of bias. Yellow: high risk, blue: low risk and grey: unclear risk.

Characteristics of included studies are listed in Table 2. Most studies were of a prospective cohort design and used pregnancy rate as outcome measure. Fig 2 and Table 3 show the scores on overall risk of bias and concerns regarding applicability in this meta-analysis according to QUADAS-2. For about half of the studies the threshold for sperm DNA fragmentation was not pre-specified and hence was judged to be at ‘high risk’ of bias for QUADAS-2 domain ‘index test’. Moreover, different cut-off values for DNA fragmentation were used to assess sperm DNA as fragmented. Studies were at high risk of applicability concerns in domain “index test” when the sperm DNA fragmentation threshold is not comparable to the thresholds of other studies. For QUADAS-2 domain ‘flow and timing’ eight studies judged to be at ‘high risk’ because of an inappropriate interval between the sperm DNA fragmentation test and the fertility treatment. Overall the reference standard was judged to be at ‘low risk’.

**Table 2:** Descriptive data of all studies for the meta-analysis regarding SCSA, SCD test, TUNEL assay and Comet assay as tools for measure sperm DNA fragmentation.

A. Characteristics of studies included in meta-analysis on SCSA							
Study	Pro-/ retrospective cohort	Infertility	Semen	Treatment	Cut-off DFI (%)	No. cycles	Outcome
Boe-Hanson 2006	Prospective	Mixed	Unclear	IVF	<27	139	CP
				ICSI	<27	47	CP
Bungum 2007	Prospective	Mixed	Pre-wash	IVF	<30	388	CP*, LB
				ICSI	<30	223	CP*, LB
Check 2005	Prospective	Previously failed ART	Unclear	ICSI	<30	106	CP, OP*
Gandini 2004	Prospective	Mixed	Pre-wash	IVF	<27**	12	P*, LB
				ICSI	<27**	22	P*, LB
Guerin 2005	Unclear	Unclear	Unclear	IVF/ICSI	<30	100	P
Larson-Cook 2003	Retrospective	Not specified	Pre-wash	IVF/ICSI	<27	89	CP, P*
Lin 2008	Pro- and retrospective	Not specified	Unclear	IVF	<9, <27*	137	CP
				ICSI	<9, <27*	86	CP
Micinski 2009	Prospective	Not specified	Unclear	ICSI	<15	60	P
Niu 2011	Prospective	Mixed	Post-wash	IVF	<27	256	CP, OP*
Oleszczuk 2016	Retrospective	Mixed	Pre-wash	IVF	<10, <20*	1117	P*, LB
				ICSI	<10, <20*	516	P*, LB
Payne 2005	Prospective	Not specified	Pre-wash	IVF/ICSI	<27	98	P
Simon 2014	Prospective	Not specified	Pre-wash	IVF/ICSI	<27	96	P
Speyer 2010	Pro- and retrospective	Mixed	Pre-wash	IVF	<19, <30*	192	P
				ICSI	<19, <30*	155	P
Virro 2004	Pro- and retrospective	Not specified	Pre-wash	IVF/ICSI	<30	249	OP
B. Characteristics of studies included in meta-analysis on SCD test							
Study	Pro-/retro-spective cohort	Infertility	Semen	Treatment	Cut-off DFI (%)	No. cycles	Outcome
Anifandis 2015	Prospective	Not specified	Pre-wash	IVF/ICSI	<35	156	CP*, OP
Ni 2014	Prospective	Not specified	Unclear	IVF	<30	855	CP*, LB
				ICSI	<30	227	CP*, LB
Muriel 2006	Prospective	Not specified	Post-wash	IVF/ICSI	<32.8	85	P
Wang 2014	Prospective	Male infertility	Pre- and post-wash	ICSI	<30	45	CP
Yilmaz 2010	Prospective	Male infertility	Pre-wash	ICSI	<30	60	P
C. Characteristics of studies included in meta-analysis on TUNEL assay							
Study	Pro-/retro-spective cohort	Infertility	Semen	Treatment	Cut-off DFI (%)	No. cycles	Outcome
Benchaib 2007	Prospective	Not specified	Post-wash	IVF	<15	88	CP, OP*
				ICSI	<15	234	CP, OP*
Borini 2006	Prospective	Mixed	Post-wash	IVF	<10	82	CP
				ICSI	<10	50	CP
Esbert 2011	Prospective	Mixed	Pre-wash	IVF	<36	77	CP
Frydman 2008	Prospective	Mixed	Pre-wash	IVF	<35	117	CP, OP*, LB
Henkel 2004	Prospective	Not specified	Pre-wash	IVF	<36.5	167	P
Huang 2005	Retrospective	Not specified	Post-wash	IVF	<4, <10, <15*	217	P
				ICSI	<4, <10, <15*	86	P
Ozmen 2007	Prospective	Not specified	Post-wash	ICSI	<4, <10*	42	CP
Seli 2004	Prospective	Not specified	Post-wash	IVF/ICSI	<20	49	CP
Simon 2014	Prospective	Not specified	Post-wash	IVF/ICSI	<10	224	P
D. Characteristics of studies included in meta-analysis on Comet assay							
Study	Pro-/retro-spective cohort	Infertility	Semen	Treatment	Cut-off DFI (%)	No. cycles	Outcome
Simon 2010	Prospective	Not specified	Pre- and post*-wash	IVF	<44, <56*	224	CP*, LB
				ICSI	<44, <56*	127	CP*, LB
Simon 2011	Prospective	Male infertility	Pre- and post*-wash	IVF	<42, <52*	70	P
Simon 2011	Prospective	Mixed	Pre- and post*-wash	IVF	<42, <52*	73	P
Simon 2014	Prospective	Not specified	Unclear	IVF/ICSI	<82	229	P

* used for meta-analysis

** threshold determined by authors of this review. CP: clinical pregnancy; DFI: DNA fragmentation index; LB: live birth; OP: ongoing pregnancy; P: pregnancy; SCD: sperm chromatin dispersion; SCSA: sperm chromatin structure assay.

**Table 3:** Study characteristics according to QUADAS II recommendations to report the risk of bias for patient selection and the concerns for applicability of data collected in manuscripts eligible for the meta-analysis.

	Risk of bias				Applicability concerns		
	Patient selection	Index test	Reference standard	Flow and timing	Patient selection	Index test	Reference standard
Anifandis 2015	low	high	low	low	low	low	low
Benchaib 2007	unclear	high	low	high	low	low	low
Boe-Hanson 2006	high	high	low	high	low	low	low
Borini 2006	low	low	low	low	low	low	low
Bungum 2007	low	low	low	low	low	low	low
Check 2005	high	low	low	high	high	low	low
Esbert 2011	low	low	low	unclear	low	high	low
Frydman 2008	low	low	low	low	low	high	low
Gandini 2004	low	low	low	unclear	low	low	low
Guerin 2005	unclear	unclear	low	unclear	unclear	low	low
Henkel 2004	unclear	high	low	high	low	high	low
Huang 2005	unclear	unclear	low	low	low	low	low
Larson-Cook 2003	low	high	low	unclear	low	low	low
Lin 2008	high	high	low	unclear	low	low	low
Micinski 2009	low	low	low	unclear	low	high	low
Muriel 2006	low	high	low	low	low	high	low
Ni 2014	high	low	low	unclear	low	low	low
Niu 2011	high	high	low	low	low	low	low
Oleszczuk 2016	low	low	low	low	low	low	low
Ozmen 2007	unclear	high	low	low	low	low	low
Payne 2005	low	low	low	unclear	low	low	low
Seli 2004	low	high	low	low	low	low	low
Simon 2010	low	high	low	unclear	low	low	low
Simon 2011	low	high	low	high	low	low	low
Simon 2011	low	high	low	high	low	low	low
Simon 2014	low	high	low	high	low	high/low*	low
Speyer 2010	low	unclear	low	high	low	low	low
Virro 2004	low	low	low	low	low	low	low
Wang 2014	low	low	low	low	low	low	low
Yilmaz 2010	low	low	low	low	low	low	low

* high risk for Comet, low risk for SCSA and TUNEL.

### SCSA

**Fig 3:**
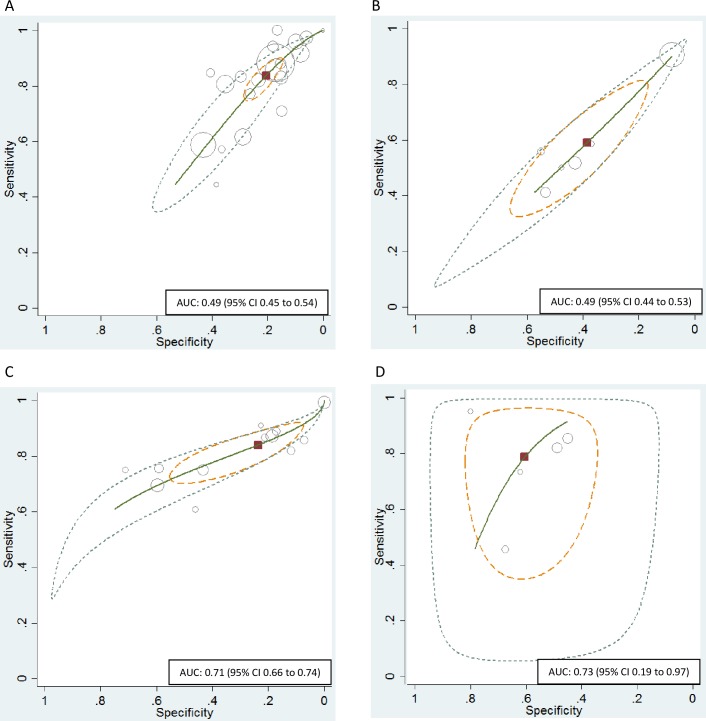
HSROC curve. Hierarchical summary receiver operating characteristic (HSROC) plot of sperm DNA fragmentation for prediction of (clinical) pregnancy. Each circle on the plot represents the pair of sensitivity and specificity from a study and the size of the circle is scaled according to the sample size of the study. The solid red block represents the summary sensitivity and specificity, and this summary point is surrounded by a 95% confidence region (yellow dashed line) and 95% prediction region (green dotted line). Sperm DNA fragmentation in the prediction of (clinical) pregnancy for all studies and all cut-off values of the DNA fragmentation index reported: (A) SCSA, (B) SCD test, (C) TUNEL assay and (D) alkaline Comet assay. AUC: Area under the curve; HSROC: Hierarchical summary receiver operating characteristics.

**Fig 4:**
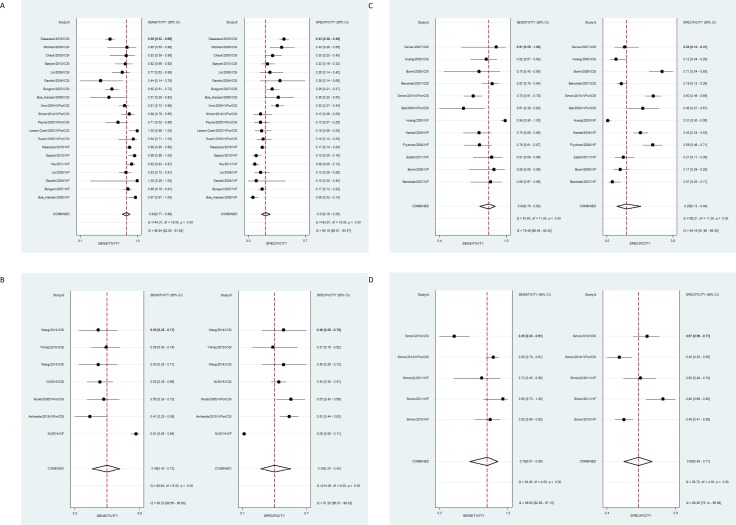
Forest plot. Forest plot of sperm DNA fragmentation according to the DNA fragmentation index for predicting pregnancy. The plot shows study-specific estimates of sensitivity and specificity (with 95% confidence intervals). The studies are ordered according to the type of treatment: (A) SCSA, (B) SCD test, (C) TUNEL assay and (D) alkaline Comet assay. CI: confidence interval.

The predictive accuracy for pregnancy with MAR of the SCSA was poor. The HSROC curve analysis indicated a sensitivity of 0.84 (95% CI 0.77 to 0.88) and specificity of 0.21 (95% CI 0.16 to 0.26) resulting in an AUC of 0.49 (95% CI 0.45 to 0.54) (Figs 3 and 4). Meta-regression indicated a difference in predictive value between IVF and ICSI (p-value: 0.00) (Table 4). For the seven studies on IVF separately the HSROC was 0.53 (95% CI 0.48 to 0.57). For the eight studies on ICSI the HSROC was 0.45 (95% CI 0.40 to 0.49). For IVF the sensitivity of the DNA fragmentation index was higher, however the specificity was lower. The low specificity points to a low proportion of true negatives, indicating low sperm DNA fragmentation does not guarantee more pregnancies. There was significant heterogeneity across studies in sensitivity and specificity (I^2^ statistic > 50%) (Fig 4, Table 4).

**Table 4:** Meta-regression analysis with type of fertility treatment as independent variable to determine whether this independent variable could be of influence on the sensitivity and specificity of the sperm DNA fragmentation test.

Sperm DNA fragmentation test	Sensitivity (95% CI)	p-value*	Specificity (95% CI)	p-value**	p-value***	I^2^
SCSA	0.69 (0.60–0.77)	0.00	0.33 (0.27–0.40)	0.00	0.00	91
SCD	-	-	-	-	-	-
TUNEL	0.79 (0.64–0.89)	0.52	0.33 (0.13–0.62)	0.60	0.59	0
Comet	0.63 (0.46–0.78)	0.08	0.60 (0.42–0.76)	0.99	0.12	52

* meta-regression sensitivity

** meta-regression specificity

*** meta-regression joint model; het-erogeneity was quantified by using the I^2^ statistic

### SCD test

The predictive accuracy for pregnancy with MAR of the SCD test was poor. The HSROC curve analysis indicated a sensitivity of 0.59 (95% CI 0.43 to 0.73) and specificity of 0.39 (95% CI 0.25 to 0.55) resulting in an AUC of 0.49 (95% CI 0.44 to 0.53) (Figs 3 and 4). There was significant heterogeneity across studies in sensitivity and specificity (I^2^ statistic > 50%) (Fig 4).

### TUNEL assay

The predictive accuracy for pregnancy with MAR of the TUNEL assay was fair. The HSROC curve analysis indicated a sensitivity of 0.84 (95% CI 0.75 to 0.90) and specificity of 0.24 (95% CI 0.11 to 0.44) resulting in an AUC of 0.71 (95% CI 0.66 to 0.74) (Figs 3 and 4). For the six studies on IVF the AUC was comparable (0.72; 95% CI 0.68 to 0.76). Meta-regression indicated no difference in predictive value between IVF and ICSI (p-value: 0.59) (Table 4). There was no significant heterogeneity across studies in sensitivity and specificity (I^2^ statistic = 0%) (Fig 4).

### Comet assay

The predictive accuracy for pregnancy with MAR of the alkaline Comet assay was fair. The HSROC curve analysis indicated a sensitivity of 0.79 (95% CI 0.61 to 0.90) and specificity of 0.60 (95% CI 0.48 to 0.71) resulting in an AUC of 0.73 (95% CI 0.19 to 0.97) (Figs 3 and 4). Meta-regression indicated no significant difference in predictive value between IVF and ICSI (p-value: 0.12) (Table 4). There was significant het-erogeneity across studies in sensitivity and specificity (I^2^ statistic > 50%) (Fig 4).

## Discussion

This current review and meta-analysis summarizes the available knowledge concerning the value of sperm DNA fragmentation tests in the prediction of pregnancy after IVF or ICSI. From the HSROC curves (Fig 3) it becomes clear that the SCSA and the SCD test have a poor predictive value, whereas the predictive value of the TUNEL assay and Comet assay was fair. All tests show higher sensitivity and lower specificity for their predictive power, indicating low sperm DNA fragmentation does not guarantee more pregnancies. Overall, there was significant statistical heterogeneity across studies (Fig 4).

We found six meta-analyses investigating the effect of sperm DNA fragmentation on live birth or pregnancy after IVF and/or ICSI. In the meta-analysis of Evenson and Wixon there was a non-significant trend towards the occurrence of pregnancy (odds ratio (OR) 1.6; 95% CI 0.92 to 2.94) when infertile couples were treated with IVF or ICSI and the DFI, determined by the SCSA, was below 30% [153]. The meta-analysis of Li et al. found that the clinical pregnancy rate decreased significantly for IVF patients with a high degree of sperm DNA fragmentation, determined by the TUNEL assay (relative risk (RR) 0.68; 95% CI 0.54 to 0.85) [154]. In ICSI clinical pregnancy rate was unaffected by DFI (RR 0.76; 95% CI 0.55 to 1.04). The meta-analysis of Collins et al. found a significant association between sperm DNA fragmentation, determined by the TUNEL assay or SCSA, and pregnancy after IVF or ICSI (OR 1.44; 95% CI 1.03 to 2.03) [155]. The meta-analysis of Zhao et al. showed that the pregnancy rate decreased significantly for IVF/ICSI patients with a high degree of sperm DNA fragmentation, determined by several sperm DNA fragmentation tests (RR 0.81; 95% CI 0.70 to 0.95) [156]. The meta-analysis of Zhang et al. showed that patients were more likely to get pregnant if DFI was less than 27% (OR 1.44; 95% CI 1.19 to 1.74) [157]. The most recent meta-analysis of Osman et al. found that the live birth rate after IVF and/or ICSI increased significantly in patients with low sperm DNA fragmentation (RR 1.17; 95% CI 1.07 to 1.28) [158]. In all the above meta-analyses the association between the sperm DNA fragmentation test and live birth or pregnancy was determined and expressed as ORs and RRs. An association does however not imply that the test actually has predictive value. Furthermore, we found several studies investigating the significance of sperm DNA fragmentation as a continuous variable in IVF and/or ICSI. These studies were excluded from meta-analysis, because no cut-off values were used so a 2x2 table could not be constructed from the data. Some of these studies found an association between sperm DNA fragmentation and pregnancy [83,92,93,97,104,107], while others did not find an association between sperm DNA fragmentation and conception [47,116]. In conclusion, we think that the best way to judge the value of DNA fragmentation tests is by their predictive capacity for the outcome of interest, ongoing pregnancy. Therefore the outcomes of our review are not comparable with the outcomes of other reviewers that chose to just report on the association between sperm DNA fragmentation and pregnancy. The results of the individual studies that were not included in our and other reviews are conflicting and lack the statistic power of a review.

Many studies investigated the predictive accuracy of sperm DNA fragmentation tests on the outcome of MAR. It is of clinical importance to assess whether these tests can be used as a prognostic tool, to distinguish couples who should be advised to undergo MAR or not. It has been suggested that DNA fragmentation is a useful marker in the prediction of spontaneous pregnancy in couples with unexplained subfertility. The chance of spontaneous conception declines at sperm DNA fragmentation index values above 20% and approaches zero for values over 30–40%. Low sperm DNA fragmentation however does not guarantee normal male fertility [159,160]. To our knowledge no study exists in which the spontaneous pregnancy chance is compared with the chance after MAR for different DNA fragmentation values.

As mentioned before, the methods of assessment of sperm DNA integrity are different for the different assays. The TUNEL assay and Comet assay are direct methods to assess DNA strand breaks, whereas the SCSA and SCD test are indirect methods, which use the higher susceptibility of damaged DNA to denature and/or fragment in an acid solution. Possibly, the fair predictive accuracy of the TUNEL assay and Comet assay, in comparison to the poor accuracy found for other methods in our meta-analysis, is due to its direct method of assessment, which may better reflect genome integrity of the sperm cells.

Besides integrity of sperm DNA, there are other factors that affect the probability to conceive after MAR, primarily the quality and age of the oocyte [161]. Male germ cells are susceptible to the accumulation of DNA lesions in fertilizing sperm because their DNA repair capacity declines during the latter part of spermatogenesis [162]. In contrast, the oocyte is capable of repairing DNA damage throughout oogenesis and provides gene products that are responsible for repairing DNA damage in both parental genomes after fertilization [163,164]. However, the competency for DNA repair depends on the quality of the oocyte which declines with age [108,161].

This review has brought forward some limitations in the available literature on DNA fragmentation tests that need to be addressed. First the study heterogeneity was high. Some studies only included couples suffering with male subfertility, other studies only included couples after previously failed MAR and others included all couples undergoing MAR. Second, the timing of performing the sperm DNA fragmentation test was not uniform. Some studies performed their test a few months before start of the MAR, others performed their test during MAR; before or after semen preparation. In addition, different cut-off values were used to assess sperm DNA as being fragmented and some studies did not even have a pre-specified threshold. However, for this reason, a bivariate model was used for the HSROC curve analysis in the present review, which allows for variations in the assessment of sperm DNA fragmentation and the choice of cut-off values. Another limitation is the reproducibility of sperm DNA fragmentation assessment. Intra-assay variability appears to be different depending on which sperm DNA fragmentation test is being used; previous studies reported this to be either small but significantly different with the TUNEL assay [165] or small and not significantly different using a alkaline Comet assay [166] and no difference using the SCSA test [32]. On the other hand, inter-observer variability was found to be very similar [21,165,167]. Mainly the TUNEL assay has many protocols, which makes comparison between laboratories hard and explains its many clinical thresholds [168]. To take care of these problems, there is need for studies that have been done with exacting protocols in the clinic and in the measuring laboratory on many patients at one center for each kind of sperm DNA fragmentation test. Unfortunately, only few studies/clinics meet these criteria [58,132,134,150–152].

It must also be mentioned that there was no correction for confounders possible. Insufficient data were available for potential confounders such as female age, male age, semen parameters and number of oocytes. Further research, for instance an IPD meta-analysis, must reveal the possible interrelation of other factors with pregnancy chance.

### Conclusions

Our systematic review and meta-analysis suggests that current sperm DNA fragmentation tests have limited capacity to discriminate between couples who have a low chance to conceive and couples who have a high chance to conceive after MAR. In addition, sperm DNA fragmentation tests have little or no difference in predictive value between IVF and ICSI. At this moment there is insufficient evidence to recommend the routine use of sperm DNA fragmentation tests in couples undergoing MAR both for the prediction of pregnancy and for the choice of treatment. Given the significant limitations of the evidence and the methodological weakness and design of the included studies, we do urge for further research on the predictive value of sperm DNA fragmentation for the chance of a spontaneous pregnancy or a pregnancy after MAR.

## Appendix

("dna damage"[MeSH Terms] OR ("dna"[All Fields] AND "damage"[All Fields]) OR "dna damage"[All Fields]) OR ("dna"[All Fields] AND "fragmentation"[All Fields] OR "dna fragmentation"[All Fields]) AND ("humans"[MeSH Terms] OR "humans"[All Fields] OR "human"[All Fields]) AND ("comet assay"[MeSH Terms] OR ("comet"[All Fields] AND "assay"[All Fields]) OR "comet assay"[All Fields] OR "comet"[All Fields]) OR ("Clin Mol Allergy"[Journal] OR "cma"[All Fields]) OR SCSA[All Fields] OR ("chromatin"[MeSH Terms] OR "chromatin"[All Fields]) OR (" acridine orange" [All Fields]) OR ("in situ nick-end labeling"[MeSH Terms] OR ("situ"[All Fields] AND "nick-end"[All Fields] AND "labeling"[All Fields]) OR "in situ nick-end labeling"[All Fields] OR "tunel"[All Fields]) OR ("in situ nick end labelling"[All Fields] OR "in situ nick-end labeling"[MeSH Terms] OR ("situ"[All Fields] AND "nick-end"[All Fields] AND "labeling"[All Fields]) OR "in situ nick-end labeling"[All Fields] OR ("situ"[All Fields] AND "nick"[All Fields] AND "end"[All Fields] AND "labeling"[All Fields]) OR "in situ nick end labeling"[All Fields]) AND ("spermatozoa"[MeSH Terms] OR "spermatozoa"[All Fields] OR "sperm"[All Fields]) AND ("pregnancy"[MeSH Terms] OR "pregnancy"[All Fields]).

## Supporting Information

S1 Fig PRISMA checklist. (DOCX)
